# Response to Leković and Nikolić

**DOI:** 10.1007/s12024-025-01008-5

**Published:** 2025-04-21

**Authors:** Alberto Amadasi, Lorenzo Franceschetti, Larissa Amadasi, Lars Oesterhelweg

**Affiliations:** 1 Institute of Legal Medicine and Forensic Sciences, University Medical Centre Charité, University of Berlin, Turmstr. 21, Building N, Berlin, 10559 Germany; 2 Department of Biomedical Sciences for Health, Section of Forensic Medicine, University of Milan, Milan, Italy

Dear Editor,

We sincerely appreciate the interest of in our work and welcome the opportunity to clarify certain aspects of the cases presented in our articles [1, 2] and the reasoning related to the possible interpretation of the fracture lines. We consider the theory of the formation of fractures associated with the temporary cavity to be potentially interesting and worthy of consideration among the possible interpretations. However, in our opinion, the data derived from our cases as well as those from the forensic literature make this hypothesis unlikely.

First of all, the suggestion that the observed fracture pattern might result from the temporary cavity effect appears partially conceptually misplaced. In one of the two cases (Case 1) of our previously published article [2] which the authors refer to, the injury was due to blunt force trauma, not a gunshot wound. As is well known, blunt trauma does not produce a temporary cavity. Therefore, invoking this mechanism to explain the fracture pattern in that specific case is simply not applicable.

Secondly, the authors imply that the fracture lines described in our cases could be attributed to the propagation of indirect fractures due to intracranial pressure rise and cavitation effects. While such mechanisms have been described in the context of high-velocity gunshot injuries, they are typically associated with fine cranial structures, such as the anterior cranial base or squamous part of the temporal bone [3–5]. The cases we reported involve fractures on robust structures, such as the parietal bones, where this type of indirect fracturing is far less likely to occur, especially in the absence of massive pressure transients typically seen with high-energy projectiles.

It is true, and this can be agreed upon, that a trepanation hole may represent a possible *locus minor resistentiae*, facilitating the formation of bone fractures secondary to the margins. However, we believe that this possibility is considerably less likely than a local deformative effect on the cranial bone surface, leading to the transmission of fracture energy (resulting from a gunshot wound) across the hole’s margins to the opposite side. A certain “symmetry” in the fracture trajectory observed on both sides of the hole in our cases also seems to support this hypothesis.

Moreover, the hypothesis proposed by Leković and Nikolić does not address the specific morphology and trajectory of the fracture lines as described and illustrated in our report [1]. The idea that a remote, cavitation-induced fracture just happened to coincide with the burr hole margins in a way that simulates a continuous fracture line originating from the exit wound lacks anatomical and biomechanical plausibility. According to the reconstruction performed by the authors, the fracture originating from the exit hole (indicated by the black arrow in their reconstruction) is said to have stopped precisely (i.e., exactly, at a very specific point, indicated by an “x” in the authors’ figure) at the end of the fracture originating from the hole, which is hypothesized to be caused by the effects of the temporary cavity. However, while we cannot exclude a priori that this may indeed be the case, such an intersection of two fracture lines appears to be somewhat “unusual” leading us to consider that it is far more likely that this represents a single fracture line originating from the gunshot exit wound, which then curves towards the craniotomy hole.

Finally, while we agree that exceptions to physical principles such as Puppe’s rule should be invoked cautiously, our case documentation, including the temporal sequence of events and the surgical context, provides strong evidence that, in rare cases, recent surgical alterations such as burr holes may act as preferential pathways for fracture propagation.

We therefore reaffirm our interpretation and the cautionary note presented in our article regarding the potential for skull fractures to traverse preexisting burr holes, potentially mimicking or obscuring the sequence of traumatic events.
